# Efficacy of convalescent plasma according to blood groups in COVID-19 patients

**DOI:** 10.3906/sag-2007-59

**Published:** 2021-02-26

**Authors:** Tuba HACIBEKİROĞLU, Yasin KALPAKÇI, Ahmet Cihad GENÇ, İlhan HACIBEKİROĞLU, Cenk SUNU, Adem SARICAOĞLU, Yakup TOMAK, Oğuz KARABAY, Mehmet KÖROĞLU

**Affiliations:** 1 Department of Hematology, Faculty of Medicine, Sakarya University, Sakarya Turkey; 2 Department of Internal Medicine, Sakarya University, Research and Training Hospital, Sakarya Turkey; 3 Department of Medical Oncology, Faculty of Medicine, Sakarya University, Sakarya Turkey; 4 Department of Blood Transfusion Center, Faculty of Medicine, Sakarya University, Sakarya Turkey; 5 Department of Anesthesiology and Pain Medicine, Faculty of Medicine, Sakarya University Sakarya Turkey; 6 Department of Infectious Disease, Faculty of Medicine, Sakarya University, Sakarya Turkey; 7 Department of Microbiology, Faculty of Medicine, Sakarya University, Sakarya Turkey

**Keywords:** Blood groups, COVID-19, convalescent plasma

## Abstract

**Background/aim:**

In this study, we aim to investigate the efficacy of convalescent plasma (CP) according to blood groups (BGs) in the treatment of critically ill patients diagnosed with COVID-19.

**Materials and methods:**

Twenty-eight critically ill and laboratory-confirmed COVID-19 patients who were admitted to the intensive care unit (ICU) of Sakarya University, Medical Faculty were included in the study. Patients were divided into 2 groups: patients who received anti-A antibody (Ab) containing CP (BG O and B) and those who did not receive CP containing anti-A Ab (BG A and AB).

**Results:**

Among the 28 patients, 13 patients received anti-A Ab containing CP (BG; B: 6, O: 7) and 15 patients did not receive anti-A Ab CP (BG; A: 13, AB: 2). Duration in ICU, the rates of mechanical ventilation (MV) support and vasopressor support, the case fatality rate, and the discharge rate were lower in patients who received CP containing anti-A Ab than not containing anti-A Ab CP. However, only the difference in the rate of MV support achieved statistically significance (P = 0.04)

**Conclusion:**

In our study, it was observed that the efficiency of CP without anti-A antibody was lower than that of plasma containing anti-A antibody, although it was not statistically significant. This result is thought to be due to the anti-A antibody’s ability to block the ACE2 receptor. We believe that this hypothesis should be investigated in controlled studies with higher patient numbers.

## 1. Introduction

The new type of Coronavirus (CoV) first appeared in Wuhan, China in December 2019 with a cluster of unknown and unidentified pneumonia [1]. After genetic analysis of the virus, it was understood that these pneumonia cases were caused by the 2019 Novel CoV (2019-nCoV), which was later called SARS-CoV-2 [2]. The disease caused by 2019-nCoV was officially named COVID-19 and was declared as a pandemic by the World Health Organization (WHO) on March 11, 2020.

Convalescent Plasma (CP) is a passive antibody (Ab) treatment that was used during the 2003 SARS, 2009 H1N1, 2012 MERS, and 2013 Ebola outbreaks and has been shown to be effective [3,4]. Based on all of these experiences, the United States Food and Drug Administration (FDA) approved CP in the treatment of critically ill COVID-19 patients on March 24, 20201FDA (2020). Investigational covid‐19 convalescent plasma—emergency INDs [online]. Website https://www.fda.gov/vaccines-blood-biologics/investigational-new-drug-ind-or-device-exemption-ideprocess-cber/investigational-covid-19-convalescent-plasma-emergency-inds [accessed 11 May 2020].. However, there are some issues that need to be clearly determined to optimize CP treatment, such as the efficacy and safety of the treatment, optimum volume of CP, the number of transfusions, the interval between transfusions, the optimum titer of neutralizing Ab, efficacy of pathogen inactivation processes, and the optimal donation time. 

In the pathogenesis, the entry gate of SARS-CoV-2 has been shown to be angiotensin-converting enzyme 2 (ACE2) receptors. SARS-CoV-2 binds to ACE2 receptors with the S glycoprotein on its surface and initiates the immune response [5,6]. A recent study has reported a structural similarity between the receptor-binding domains of SARS-CoV and SARS-CoV-2 [7]. Researchers revealed that natural plasma anti-A antibodies inhibited the SARS-CoV S protein/ACE2-dependent adhesion to ACE2-expressing cells [8]. Since SARS-CoV2 uses the same receptor as SARS-CoV, anti-A isoagglutinins are expected to have similar effects against SARS-CoV2 [9]. Anti-A natural isoagglutinin can be also found in CP donated from O and B blood group (BG) donors. Therefore, it can be inferred that the treatment results of O and B BG patients may be better as a result of the natural presence of anti-A antibodies and administration of extra in CP. In this study, we aim to investigate the efficacy of CP according to BGs in the treatment of critically ill patients diagnosed with COVID-19.

## 2. Materials and methods

### 2.1. Patient population

Approximately 28 critically ill and laboratory-confirmed COVID-19 patients who were admitted to the intensive care unit (ICU) of Sakarya University, Medical Faculty between March 11, 2020 and June 02, 2020 and who received a CP transfusion in addition to antiviral therapy were included in the study. All patients were detected to be SARS-CoV-2 RNA positive by nasal and pharyngeal swab samples using a SARS-CoV-2 nucleic acid detection kit (Genesig Real-Time, Primer Design, UK). Critical COVID-19 was defined as exhibiting the presence of respiratory failure, septic shock, and/or multiple organ dysfunctions1. Therapeutic apheresis centers licensed by the Republic of Turkey’s Ministry of Health and the Turkish Red Crescent carried out activities to obtain CP from donors. Donor assessments were performed according to the Republic of Turkey’s Ministry of Health and Donor Eligibility Criteria for COVID-19 Convalescent Plasma2Turkish Ministry of Health (2020). COVID-19 İmmün (Konvalesan) Plazma Tedarik ve Klinik Kullanım Rehberi [online]. Website https://dosyamerkez.saglik.gov.tr/Eklenti/37163,covid-19-immun-plazma-rehberi-12-nisan-2020-sonv1-ti-neopdfpdf.pdf?0 [accessed 17 April 2020].. In addition to nucleic acid amplification tests, all donors were serologically screened for HBsAg, anti-HCV, anti-HIV 1-2 and, anti-Syphilis Ab. The titer of neutralizing Ab was not routinely performed. CP was collected by apheresis. Approximately 200–600 cc of plasma was collected with apheresis devices depending on the total blood volume of the donor. Plasma components were labeled using the ISBT128 coding system and stored at or below –18/25 C° in storage cabinets. Pathogen inactivation processes were not routinely performed. Patients were divided into 2 groups as anti-A Ab containing CP received patients (BG O and B) and those who received CP not containing anti-A Ab (BG A and AB). Clearance of SARS-CoV-2 was defined as at least 2 consecutive negative RT-PCR test results.

Local Ethical Committee approval was obtained from Sakarya University, numbered 71522473/050.01.04/342 and, additionally, Turkish Health Ministry approval was also obtained on June 12th, 2020 for this study, as required.

### 2.2. Statistical analysis

IBM SPSS v26 software (IBM Corp., Armonk, NY, USA) was used for analysis. Descriptive statistics were implemented to summarize data. Variables assessed for normal distribution with graphics and the Shapiro–Wilk test. An independent t-test was applied to assess age and ICU duration between groups Categorical data were presented as number and percentages, and numerical data were presented as mean ± standard deviation. Differences between categorical variables were analyzed with the chi-square test, and parametric continuous variables were analyzed with a one-way ANOVA test. A two-sided P-value ≤0.05 was considered statistically significant.

## 3. Results

Among the 28 patients included in this study, 13 patients received anti-A Ab containing CP (BG; B: 6, O: 7), and 15 patients received CP not containing anti-A Ab ( BG; A: 13, AB: 2). The characteristics of the patients are given in Table 1. All patients received favipiravir, lopinavir/ritonavir, and hydroxychloroquine. Eleven patients received 3 CP transfusions (600 cc), 10 patients received 2 CP transfusions (400 cc), and 7 patients received 1 CP transfusion (200 cc). When we compared the outcome of patients who received CP containing anti-A Ab to the outcome of patients who received CP not containing anti-A Ab, the duration in the ICU was shorter. In addition, the rates of mechanical ventilation (MV) support and vasopressor (VP) support, the case fatality rate (CFR), and the discharge rate were lower in patients who received CP containing anti-A Ab. However, only the difference in the rate of MV support achieved statistical significance (P = 0.04) (Table 2). When BGs were analyzed separately, no statistically significant difference was observed regarding the duration in ICU, the rates of MV support and VP support, and the CFR and discharge rates.

**Table 1:** Characteristics of patients.

	A+ AB(n = 15)	B+O(n = 13)	P value
ICU stay (mean±SD)	15.3 ± 7.7	12 ± 9.8	0.62
MV support	14 (93.3%)	8 (61.5%)	0.04*
VP support	12 (80%)	6 (46.2%)	0.06
Discharge rate	4 (26.7%)	6 (46.2%)	0.28
CFR	9 (60%)	7 (53.8%)	0.74

SD: standard deviation; N/A: not applicable.

**Table 2:** Outcome of patients.

Characteristics	A and AB(n = 15)	B and O(n = 13)	P value
Sex Male: n (%) Female: n (%)	9 (60%) 6 (40%)	11 (84.6%) 2 (15.4%)	0.22
Age, years (mean±SD)	64.6 ± 12.3	62.6 ± 9.8	0.75
Comorbidity: n (%) Diabetes mellitus Hypertension Cardiovascular diseases Respiratory system diseases Chronic renal diseases Cerebrovascular diseases Cancer	7 (46.7%) 9 (60%) 3 (20%) 1 (6.7%) 1 (6.7%) 0 1 (6.7%)	5 (38.5%) 5 (38.5) 2 (15.4%) 0 1 (7.7%) 1 (7.7%) 0	N/A

CFR: case fatality rate; ICU: intensive care unit; MV: mechanical ventilation; VP: vasopressor support; SD: standard deviation.

## 4. Discussion

Prediction of ABO BGs has been previously investigated, especially in viral infections such as HIV, Hepatitis B, and Severe Acute Respiratory Syndrome (SARS) [10]. In a previous study, anti-A Ab was shown to neutralize HIV produced by lymphocytes from blood group A donors only [11]. Similar to the reports about HIV, researchers demonstrated that the measles virus was neutralized by natural anti-A Ab in a complement-dependent manner [12]. If natural anti-A or -B serum antibodies provide protection, it is expected that during an outbreak, BG O individuals should experience a lower risk of infection than non-BG O individuals. During the outbreak of SARS, the O BG group was reported as having a lower risk of being infected by SARS-CoV than subjects from other BG groups [8,13]. SARS-CoV infects cells such as pneumocytes, enterocytes of the small intestine, as well cells of the kidney distal tubular epithelium—all cell types known to be able to synthesize ABH antigens [14,15,]. There are studies in the literature reporting that there is a structural similarity between the receptor binding domains of SARS-CoV and SARS-CoV-2. The main mechanism of SARS-CoV-2 is that it binds to human ACE2 surface receptors with the S glycoprotein on its surface and enters the cell and initiates immune mechanisms [16]. 

Anti-A Ab was shown to inhibit the SARS-CoV S protein/ACE2-dependent adhesion. In addition to the blocking of virus attachment to its receptor, natural antibodies can block entry or opsonize viral particles leading to complement-mediated neutralization [17]. A recent publication showed that the odds ratio for acquiring COVID-19 is higher in blood group A than in blood group O [18]. Elderly males are known to experience reductions in isoagglutinin titers, and previous studies have shown that COVID-19 has more severe clinical presentations and outcome in elderly males [19,20]. On the other hand, plasma VWF levels are 20%–30% lower in normal blood group O individuals compared to non-O subjects [21]. Since FVIII circulates in high-afﬁnity complex with VWF, plasma FVIII levels are also reduced in blood group O individuals. The reduced risk of COVID-19 induced coagulopathy, due to the lower level of FVIII/VWF in O BG patients, may be another hypothesis that could explain the positive clinical course [22]. In our study, when we compared the outcome of patients who received CP containing anti-A Ab to the outcome of patients who received CP not containing anti-A Ab, the duration in the ICU was shorter and the rates of MV support and VP support and CFR and discharge rates were lower in patients who received CP containing anti-A Ab. However, only the difference in the rate of MV support achieved statistical significance. Although there are studies in the literature examining the predictive effect of BGs on COVID-19 patients, there is no study investigating this relationship in critically ill COVID-19 patients receiving CP. The opinion that Focosi stated in his letter that CP containing high titer anti-A isoagglutinin may be more effective in CP treatment supports the results of our study [23]. Studies are ongoing to evaluate the correlations between isoagglutinin titers and the outcome in BG 0 and B patients. 

The limitation of this study is that the number of patients is insufficient to demonstrate the statistical effect of CP on clinical results. If the number of cases were higher, visible results would be statistically significant.

In conclusion, this study shows that treatment with anti-A Ab containing CP may be more effective due to the mechanism of blocking ACE2 receptors with the anti-A Ab. Therefore, it may be more beneficial to choose the O and B blood group donors/patients and titrate anti-A isoagglutinins in the treatment of ABO blood group-compatible CP in COVID-19 patients. Larger prospective multicenter studies may be needed to demonstrate the preventive and therapeutic role of Anti-A Ab.

## Funding source

No funding was secured for this study.

## Financial disclosure

The authors have no financial relationships relevant to this article to disclose.
